# Acceptability and barriers to implementation of N-of-1 tests in Ethiopia - a qualitative study

**DOI:** 10.1186/s12874-019-0832-7

**Published:** 2019-10-15

**Authors:** Chalachew Alemayehu, Geoff Mitchell, Jane Nikles, Abraham Aseffa, Alexandra Clavarino

**Affiliations:** 1 Faculty of Medicine, The University of Queensland, P.O. Box: 78, Scott road, Brisbane, QLD 4006 Australia; 2 UQCCR, The University of Queensland, Brisbane, Australia; 3 Armauer Hanson Research Institute, Addis Ababa, Ethiopia; 4 School of Pharmacy, The University of Queensland, Brisbane, Australia

**Keywords:** Generic drugs, Lack of bioequivalence data, N-of-1 bioequivalence trials, Therapeutic equivalence, Acceptability, Institutional review board, Drug regulation authority

## Abstract

**Background:**

Locally produced generic drugs offer a cost–effective alternative to imported drugs to treat patients in Ethiopia. However, due to a lack of bioequivalence testing, additional assurance tests are needed to build trust in cheaper, locally made drugs. By testing bioequivalence of local drugs to gold standard, N-of-1 tests have the potential to promote patient centred quality use of medicines.

**Method:**

We sought to assess the acceptability of, and explore barriers to, conducting N-of-1 tests to evaluate local medicines in a resource limited clinical setting. We conducted a descriptive qualitative study, analysing four focus group discussions and five key informant interviews. Participants were senior drug regulatory authority members, institutional review board members, physicians and patients. All interviews were audio taped and transcribed verbatim. Patient interviews were conducted in Amharic and translated to English prior to analysis. Data analysis used an inductive, thematic process.

**Results:**

Five major themes were identified; (1) Appropriateness of N-of-1 tests to determine the therapeutic equivalence of local drugs, (2) N-of-1 therapeutic equivalence tests: clinical care or research? (3) Ethical and regulatory requirements (IRB), (4) Potential barriers to implementing N-of-1 tests and (5) Possible solutions to identified challenges. The study demonstrated considerable support for using N-of-1 tests for clinical equivalence studies between local and imported medicines, but important impediments were very likely to impact the feasibility of conducting N-of-1 tests in Ethiopia. Key informants from the regulatory authority did not support additional tests of local drugs. There were also mixed opinions regarding ethical requirements for conducting N-of-1 tests. The Institutional Review Board (IRB) members believed that IRB approval was sufficient to conduct N-of-1 tests, however, the regulatory authority members considered that N-of-1 tests constituted a clinical trial, and required approval at the regulatory level.

**Conclusion:**

This study showed that there were key uncertainties that could impact the feasiblity of using N-of-1 testing local drugs in Ethiopia. Therefore, a number of protocol amendments to address contextual threats and regulatory challenges, would be needed before progressing to conducting these tests.

## Background

Ethiopia is the second most populous country in Africa with a population of approximately 100 million [1] in 2015. Non-communicable diseases disproportionately affect developing countries, including Ethiopia [2] and there is limited capacity for people to access affordable treatments [3]. Generic drugs represent a potentially cost–effective solution to treating non-communicable diseases. The United Nations recommends that the world’s poorest countries improve access to medicines through local production of generic drugs which are bioequivalent to brand-name drugs [4]. Around 30% of the National Essential Drugs in Ethiopia are produced locally [5].

The existence of rigorous drug approval and monitoring systems in the developed world has made generic substitution more reliable [6]. However, lack of sufficient medicine regulation in sub-Saharan Africa (including Ethiopia) poses difficulties in guaranteeing effective generic drug substitution [7, 8].

### Lack of a bioequivalence centre in Ethiopia

Ethiopia is one of the Sub-Saharan African countries where the poor regulation of medicines poses a challenge to the effective clinical care of patients [7, 9]. For example, bioequivalence tests, which are taken for granted in many countries, are not available [7]. Bioequivalence with reference drugs forms the basis for approval of generic drugs [10–12]. While bioequivalence certification is a requirement for registration of medicines in Ethiopia (in principle) [13], the lack of a local bioequivalence testing facility has made enforcing bioequivalence onto local pharmaceutical companies difficult. Hence, locally made medicines are marketed without fulfiling the World Health Organization-prequalification criteria for therapeutic interchangeability [7].

There is one documented case in which the Ethiopian medicine regulatory authority banned the production of one locally manufactured drug following receipt of claims of ineffectiveness of the drug from multiple health professionals [14].Though there is limited evidence about the views of local physicians on locally manufactured drugs, in one survey only 46% of prescribers agreed that locally produced and imported medicines were therapeutically equivalent [15].

Interventions that enhance the acceptance of generic drugs are highly desirable for promoting effective generic drug substitution [16–20]. For example, the US Food and Drug Authority advises research (eg. individual patient brand-to-generic switching studies) to obtain additional data in therapeutic areas where concern exists about the substitutability of generic drugs [21]. In the absence of bioequivalence data, additional tests that evaluate therapeutic equivalence are needed to ensure that patients have the option to substitute cheaper local drugs confidently and to access effective and high quality treatment in Ethiopia.

Clinical endpoint bioequivalence studies test the clinical bioequivalence of a medicine in patients [22, 23]. They can be used to establish the bioequivalence of drug products when pharmacokinetic studies are not possible [13]. Quality of care can be improved significantly by increasing the likelihood that health care providers practice in ways most beneficial to patients under the prevailing circumstances (process improvement) [24–26]. Thus, quality improvement tools must be consistently shared among developing countries to build local capacity [24, 27, 28].

### N-of-1 tests

N-of-1 tests are double blinded, multiple cycle crossover trials, comparing a test treatment with a comparator [29]. Guidelines commissioned by the US Agency for Healthcare Research and Quality (AHCRQ), and a recently published book have both documented the use of N-of-1 tests as a means of formally assessing the bioequivalence of generic drugs [30, 31]. N-of-1 tests have been used to prove the therapeutic interchangeability of generic drugs with brand drugs [20, 32].

By establishing bioequivalence information for local drugs, N-of-1 tests have the potential to provide affordable, patient-centered care and promote quality use of medicines. Compared with pharmacokinetic bioequivalence tests, N-of-1 therapeutic equivalence tests require less technology and can be done at the point of care.

This study is designed to determine the acceptability of N-of-1 tests as one approach to assessing the bioequivalence of untested, but approved, generic drugs in use in a resource limited setting. Developing interventions without adequate consideration of how the target population will accept them puts their implementation at risk [33, 34]. The context in which an intervention study is conducted is also important. Contextual threats to trial conduct are often subtle, idiosyncratic and complex, and are therefore best explored using qualitative research [35]. Qualitative research incorporated within a feasibility study allows the exploration of contextual uncertainties as well as the acceptability of the design and the intervention to be evaluated [36]. Obtaining the views of relevant stakeholders, who might take part in the study, and officials from the ethics and regulatory authorities who may need to approve proposed interventions, is a critical component for successful implementation [37–40].

This paper presents a part two of a study using qualitative research. A previous paper reported themes that related to the participant’s views on the quality and acceptability of locally produced generic medicines [41]. This paper reports participant’s views on the acceptability of N-of-1 therapeutic equivalence tests to provide bioequivalence information of untested generic drugs in a resource poor country. This paper addresses the following research questions:

- What are the views of stakeholders regarding the use of N-of-1 tests as a means of assessing the interchangeability of generic and brand name drugs in Ethiopia?
- What are the practical barriers to conducting N-of-1 tests in Ethiopia?

## Method

### Setting

The study was conducted in the All Africa Leprosy, Tuberculosis and Rehabilitation Training (ALERT) center, a medical research center which comprises ALERT hospital and the Armauer Hanson Research Institute (AHRI) in Ethiopia. The study was approved by The University of Queensland ethics review committee (approval number: 2016-SOMILRE-0158) in Australia and by the ALERT/AHRI ethics review committee locally (approval number: PO28/16). The research methods are described in detail in the previous paper (REF), and reiterated here.

### Description of the proposed intervention/clinical tests

The project introduces N-of-1 therapeutic equivalence tests in clinical practice to test local generic medicines used for the management of non-communicable diseases. Hypertension is the single most important cause of mortality from non-communicable diseases in Ethiopia and Enalapril is the most commonly used first line treatment option. Therefore, we chose Enalapril for hypertension as the clinical scenario to pilot N-of-1 tests at the point of care. This acceptability study will determine whether N-of-1 studies can be used in a developing country to identify affordable drugs that are proven to work in the individual, thereby improving clinical decision making and medicine compliance. The proposed N-of-1 test will be piloted in a research institute-affiliated hospital in Ethiopia.

### Design and data collection methods

This qualitative study was conducted to inform future implementation of the proposed N-of-1 tests of local drugs. Data were collected in April and May, 2016. Semi-structured focus group discussions and key informant interviews were undertaken (see Additional file 1). The principal investigator, along with a trained facilitator, collected data using an audio tape-recorder and field notes. Data collection was conducted in the local language (Amharic) then transcribed and translated into English. The principal investigator is an Ethiopian and native Amharic speaker and did the transcriptions.

The following issues were explored during the group discussions and interviews:

- What is your overall impression regarding the use of N-of-1 trials as a bioequivalence method to test therapeutic equivalence of local medicines? Are they appropriate? What are the advantages and disadvantages?
- How N-of-1 bioequivalence method be treated? Should it be considered as a clinical care tool that advance clinical care of patients or clinical research tool? Why?
- What are the appropriate approval and oversight requirements for conducting the N-of-1 bioequivalence study? Do you think IRB approval and oversight suffice? Do you suggest the need for regulatory approval and oversight? Why?
- What do you think are the potential barriers to conducting the proposed N-of-1 bioequivalence study?

### Participants and recruitment methods

Four focus group discussions, two with physicians and two with patients who had controlled hypertension (one comprising male patients and one comprising female patients) were undertaken. The number of participants in each focus group ranged between six and eight. Five key informant interviews were also conducted. Three were with institutional review board (IRB) members and two were with drug regulatory authority employees. Participants in the IRB group were members of the ALERT/AHRI Ethics Review Committee (AAERC). AAERC is an experianced ethics review committee that operates from the ALERT center. The two key informants representing the regulatory authority were senior officials involved in the approval and oversight of clinical trials in Ethiopia.

A purposive sampling method was used to ensure inclusion of differing perspectives from the different stakeholders who would be involved during the pilot implementation of N-of-1 tests. Physicians were purposively selected based on their position and experience in ALERT chronic care services. Because of our intention to pilot N-of-1 tests in hypertension, only hypertensive patients, who were being treated at the ALERT hospital, were invited to participate in the patient focus group discussions.

### Educational material and education sessions

Because the idea of N-of-1 tests is a new concept in the Ethiopian context, pre-reading material, which explained the rationale for the study, the proposed N-of-1 tests and their implementation strategies, was circulated to all participants before conducting discussions and interviews. The educational material covered a number of topics including bioequivalence, local generic drugs and N-of-1 tests and their implementation. The content of the material provided to the different groups of participants was adapted to meet the specific needs of each group (see Additional file 2).

Before conducting the focus group discussions, education sessions (with questions and answers) were held to enhance participants’ understanding of N-of-1 tests and the proposed study, with the intention being to have the focus group discussion focus on the research questions rather than be side-tracked by an explanation of the method.

### Data collection and analysis

Interviews lasted between 24 and 45 min and focus groups lasted from 35 to 104 min. The focus group discussions and three of the key informant interviews were audio taped and transcribed verbatim. The two senior key informants from the regulatory authority did not consent to being recorded but did agree to be interviewed. Brief notes were taken during these two interviews. On completion of each interview these notes were then expanded. The expanded field notes were then incorporated with the transcriptions to facilitate generation of themes.

After familiarisation with the data through repeated reading of the transcripts, a thematic framework [42] was developed using emerging ideas from data collection and a priori questions drawn from the objectives of the study. Five major themes were identified; (1) Appropriateness of N-of-1 tests to determine the therapeutic equivalence of local drugs, (2) N-of-1 therapeutic equivalence tests: clinical care or research? (3) Ethical and regulatory requirements, (4) Potential barriers to implementing N-of-1 tests and (5) Possible solutions to identified challenges. Transcripts were open coded for themes, a sub-set of transcripts and field notes was also coded by a second author and any discrepancies were discussed and resolved by all authors.

## Results

As reported previously [41], this study involved 31 participants: five key informant interviews (two senior regulatory authority members and three IRB members) and 26 participants (14 physicians and 12 patients) in focus group discussions. Participants ages ranged from 32 to 65. Eleven of the 26 participants were female (see Table 1). While two thirds of male patients had formal education, only one third of female patients had any formal education. Of the three IRB committee members, one was a senior physician, one was a senior researcher and the third one had an administrative role at a government health office.

**Table 1:** Characteristics of study participants

Participants	Focus group	No. of people	Gender (Male/Female)	Age	Education
Physicians	1	6	6/0	35–59	Attend tertiary education (6)
	2	8	6/2	32–54	Attend tertiary education (8)
Patients	1	6	6/0	51–60	Attend tertiary education (2) Secondary school (3) Primary School (1)
	2	6	0/6	48–57	Primary School (2) Illiterate (4)
Ethics and regulatory authorities	Key Informant I interview	Overall (5) Ethics (3) Regulatory (2)	2/3	3 36–56	Attend tertiary education (5)

Descriptive extracts of the data, categorized into the five themes, are presented below. For each quote, the unique identification number of that participant, as well as the data collection technique used, are given in brackets.

### Theme 1: appropriateness of using N-of-1 tests to determine the therapeutic equivalence of local drugs

There was a considerable amount of support for the N-of-1 approach from the different groups of participants. Almost all believed the study was an important way to identify better drugs and provide better patient care. Their impression was that N-of-1 tests could be a pragmatic solution to address uncertainties in the clinical care of patients. In particular, there was considerable support from IRB members and also from focus group participants.

*‘It (N-of-1) is really a great strategy to check the comparative effectiveness of our drugs’ (P3, IRB interview)*

*‘I am really very happy. Such kind of study has a lot of contribution to check drugs produced in Ethiopia.’ (P1, male FGD)*

Physicians and stakeholders could see the benefits for patients. They noted that identifying ineffective drugs and selecting drugs that work for individual patients are key components of providing better patient care.

*‘It is good to rule out the ineffective drugs. This project is good to differentiate which drug is really useful for the patient. It is all about the patient. I think this study is good and is useful for the patient’ (P1, physician FGD 2)*

*‘When a patient claims that a drug is not working, the doctors must consider such studies. … I think it will help to successfully treat those patients who are not benefited from the drug both physically and psychologically. (P2, IRB interview)*

N-of-1 tests were considered important as one method for potentially promoting the use of less costly drugs. Changing the mindset of physicians to show that cheaper drugs could be as effective as expensive ones could overcome an important barrier to providing cost effective treatment options for patients. Tests that compared treatment effects of different brands of drugs were identified as particularly influential in addressing this challenge, thus assisting physicians to decide which generic brands to prescribe.

*Oftentimes, physicians prescribe drugs imported from Europe but most people couldn’t afford them. There is a big difference in price among different brands of the same drug … .I think this project is helpful to prescribe cheaper drugs as long as they have the same effect as the expensive drugs.* (P3, IRB interview)

There was also recognition that N-of-1 tests could benefit physicians in somewhat unexpected ways, for example as one physician said.

*‘Actually, we physicians will benefit more from the study. We will develop our research experience and we could improve the care of our patients’ (P5, physician FGD 1)*

There was also some indication that these tests might improve the quality and drug options available in the local market.

*‘The study may encourage our local companies to improve their quality and we will have options of many local drugs with affordable costs.’ (P1, physician FGD 1)*

N-of-1 tests could also have an impact on the physician – patient relationship. Several physicians warned that testing the comparability of local drugs against imported ones could negatively affect the patient-physician interaction if the local drug was shown to be less effective. This is because those patients who could not afford the more costly drugs might lose their faith in their physician.

*‘There will be some obstacles to run this project in our country … our participants wouldn’t be able to buy alternative drugs because of financial problems. If we follow a chronic patient with one drug (generic) in N-of-1 trial and if the drug doesn’t work, the patients could lose his confidence upon the physician, not only on the drug’ (P6, physician FGD 1)*

However, it also raised an important issue regarding the implications of using ineffective drugs.

*‘If the local company produce a drug with 40% effectiveness while the [imported] brand is 80% effective, prescribing the local drug means doing nothing. Even if the patient couldn’t afford it, we may use other options like government exempted services. This all could be solved with studies like this. If the drug we prescribed is not effective we are doing futile exercise. … our patients are complaining against the practitioners, not the drugs. Therefore the study could minimize these gaps’ (P2, physician FGD 1)*

A small number of key informants did not support testing local drugs. One indicated that the authority approves effective and safe medicines, and claimed that such studies could have negative consequences for patients, companies and the regulatory authority itself.

### Theme two: N-of-1 therapeutic equivalence tests: clinical care or research?

The issue of how best to conceptualise N-of-1 tests at the point of care, that is whether they need to be treated as a clinical care tool, as clinical research or as a conventional clinical trial, was raised. The distinction between what constitutes clinical care, clinical research or a clinical trial was clear to the participants. It should be noted that patient participants were not asked questions regarding theme two. Whether N-of-1 studies constituted clinical care, research or a clinical trial was an important issue, because this would affect the need for official approval from both the ethics and regulatory authorities, and in turn, influence physicians’ use of N-of-1 tests in their daily clinical practice. There was considerable diversity of opinion from the participants.

The majority of stakeholders (including IRB members) considered that N-of-1 tests could be both a clinical care tool and clinical research. While the purpose of the test was considered to be clinical care, the processes and procedures involved (randomisation, blinding and the way outcomes are selected, assessed and reported) were considered to be clinical research..

*‘ … since it involve a randomization, blinding and recording of results, and using results as an outcome, it is a research tool. But we can see the research in a different way. What you are doing has some clinical care component.’ (P 3, IRB interview)*

*‘ … we can take it as a part of clinical care because the doctor makes his own research as to whether the drug is applicable or not. … It has also a clinical research character because we are intentionally introducing/changing the drugs. … Though it is used for clinical care purpose, I think it will also be considered as clinical research.’ (P4, physician FGD 2)*

By contrast, several considered this test only to be a clinical care tool. The type of study (involving only one participant), the study setting and parties involved (patients and physicians) and its purpose (collecting data to improve patient’s own care) were the basis for making this distinction.

*‘From the study type, it is more of a clinical care. Because only one participant is involved and data is taken from the participants and the beneficiary are the participant themselves. So, I think it’s clinical care.’ (P2, physician FGD 2)*

*‘I also believe that is about clinical care improvement. It is an interaction between the patient and the physician, not beyond. … We need to use many options to manage our patient. So that it is a clinical care not a research.’ (P1, physician FGD 1)*

key informants of the regulatory authority, however, considered N-of-1 tests in individual patients to be clinical trials. They noted that, according to the Ethiopian medicine regulations, irrespective of the degree of risk associated with the study and whether the drugs are approved for use or not, any comparative study involving drugs in humans is considered to be a clinical trial. They noted that the proposed N-of-1 tests do not satisfy the definition of standard clinical care in Ethiopia as they could involve the testing of different drugs sequentially in one patient.

### Theme three: ethical and regulatory requirements

Whether N-of-1 tests require ethical approval depended on the intention of the test; that is, whether the aim was to improve clinical care or to conduct clinical research. Ethical approval was only needed if the intention of the test was to generate generalisable data. N-of-1 tests often test drugs with established efficacy and safety profiles based on clinical trials.

Key informants from the IRB and the regulatory authority were asked whether IRB approval was sufficient or whether additional regulatory approval was required as in the case of classical clinical trials. Respondents believed that IRB approval was sufficient. One respondent also suggested that requirements should be strict as the method represents a new approach in the country.

*‘I don’t think you need this (regulatory approval). Because these drugs are already registered by Food, Medicine and Health Administration and Control Authority (FMHCA) and are being used nationally. It is a registered drug, you are not going to import it. People are [already] using it, so I don’t think it requires a strict clinical trial requirements. But still there are some trial components like randomization and blinding. So it needs IRB approval.’ (P 3, IRB interview).*

The use of the term ‘trial’ could also be a sensitive issue.

*‘My fear would be just the name trial.’ (P1, IRB interview)*

Not surprisingly, key informants representing the regulatory authority believed that the proposed study using N-of-1 tests would be considered to be a clinical trial and hence would require approval at the regulatory authority level. Although they expressed their view of the regulatory requirements clearly, these participants were unwilling to discuss and explain the ethical procedures they felt needed to be taken. They noted that they would not discuss and comment on future applications and that all applications should follow the conventional route of application - ie ethical approval and then application to the regulatory authority.

### Theme four: potential barriers to implementing the study

The participants were asked about challenges they could see arising when implementing the project. They responded from a number of perspectives.

Most physicians mentioned concerns about work load and lack of time in their daily practice. Some mentioned lack of experience with research and changing the mindset of physicians about research (some physicians have already developed an impression that research has no direct benefit in their daily patient care).

*‘Especially the issue of physician’s time, work burden and patient’s condition should be assessed. We are not also aware of research.’ (P2, physician FGD 1)*

*‘One thing I want to add is that physicians are busy and the mentality is not often good for research.’ (P6, physician FGD 1)*

Several possible barriers for the approval and conduct of the study were identified. For example, N-of-1 tests were new for the country, there was, therefore a lack of experience and framework to decide how to monitor the study.

*‘IRB will focus on the advantage from the patient perspective. It might be a bit hard for you. The IRB may want to make strict follow up as it (N-of-1) is new. Such studies are not common, so the practicability of the project needs to be given due attention.’ (P2, IRB interview)*

Identifying participants suitable for inclusion in the trial was also raised as an issue. Patients’ suspicions about the study, lack of interest in drug switching and low patient literacy potentially limited patients’ understanding of the study process. The extent of their capacity to record their own blood pressure measurements could also represent a challenge for the study.

*‘Patients in our set up are suspicious, so they might not take the drug.’ (P3, physician FGD 1)*

*‘If the patients have to record their BP by themselves, they might be illiterate.’ (P7, physician FGD 2)*

Patients’ focus was on lack of time and on financial issues, as well as logistic problems related primarily to transport. This is because many patients struggle to support their own as well as their family’s needs. Therefore, they cannot afford additional transportation, medical and other related costs that might be involved in being participating in such studies.

*‘We are busy at home for our daily life and don’t forget that we are poor.’ (P6, Female FGD)*

*‘We may have shortage of time and transportation problem.’ (P6, male FGD)*

The patient groups were also asked if they would be willing to participate in the coming N-of-1 test. The majority of the patients reported they would participate in the pilot trial if invited.

*‘I need to be a participant of the research since the finding will help me to find better drug’ (P4, male FGD)*

The sensitivity of the disease chosen was also raised as a potential barrier. One participant was concerned about the disease selected for piloting N-of-1 therapeutic equivalence tests. Because of lack of bioequivalence testing, substitution of local drugs could represent a risk for patients with hypertension if ineffective.

*‘What I want to say is that hypertensive patients need to take their drug properly. If you give a drug which is useless, blood pressure cannot be controlled, so there will be subsequent result if you don’t control the blood pressure. A patient might develop cerebral hemorrhage after taking drug which is not effective.’ (P3, IRB interview)*

### Theme five: possible solutions to address the challenges

After discussion of problems that could impact the acceptability and feasibility of the proposed pilot trial, participants identified potential solutions that could address the ethical and operational barriers that had been identified. Three strategic approaches were identified.

#### Doctor related strategies

For physicians it was found that the selection of interested physicians, incentives for participation, and well-coordinated training for trial staff would enhance participation.

*‘Priorities for training … we can easily notice the importance of training … It is also good to incentivize those professionals who participate in the study.’ (P6, physician FGD 1)*

*‘There are a lot of things, but the physician should involve themselves and devote their time and the project should consider incentives and train them as well.’ (P3, physician FGD 2)*

#### Ethics and regulatory strategies

Here it was suggested that developing a workshop or training package to enhance awareness of the study and its methodology, preparation of a standard protocol and case report forms, and the involvement of senior physicians during the planning and conduct of the trial would enhance acceptability of the proposed study.

*‘To minimize that kind of hassles as a researcher and applicant, I advise you to call for a three or four days of workshop for those who are working in the regulatory bodies and brief what your study is all about’ (P1, IRB interview)*

*‘Regarding the ethical process, it might take time, you need to think thoroughly and prepare the protocol well … the follow up needs to be rigorous. You might need to involve seniors for patient follow up’ (P3, IRB interview)*

#### Patient related strategies

Suggested strategies to address participant challenges included compensation for transport to the hospital for visits relating to the test, promoting awareness of the project and the provision of free drugs.

*‘We will participate in the study if you consider transportation costs and something to buy bread for our children when we get back to home from hospital visit’ (P2, female FGD)*

*‘Firstly, awareness creation regarding the benefit of the study for patients is important.’ (P6, male FGD)*

## Discussion

A qualitative study, using focus group discussions and key informant interviews, was conducted to assess the acceptability of N-of-1 tests as a practical, low cost means of assessing therapeutic equivalence of local generic medicines used to treat non-communicable diseases in individual patients in Ethiopia.

Most participants supported the concept of using N-of-1 tests to assess local generic medicines, and highlighted important concerns about using this approach when testing locally manufactured drugs in Ethiopia. Respondents across categories highlighted several benefits of N-of-1 tests: their capacity to potentially identify better drugs that cost less, to check the efficacy of drugs, to improve the doctor-patient relationship, to improve physician research skills and to address issues related to branding of drugs. These potential benefits have also been identified by previous researchers [30, 31].

As the major purpose of the study was to explore key uncertainties associated with conducting the study, we have summarised the learnings from this study to inform any future proposals (see Table 2). The subsequent sections discuss threats to feasibility; major threats are discussed first, followed by issues that represent minor threats.

**Table 2:** Key insights from the qualitative research on future N-of-1 therapeutic equivalence tests on local generic drugs

Area of uncertainties		Sub-category	The learnings/what should be done on the proposed test
Intervention		Regulatory authority members did not support the need for testing local drugs	This is a key uncertainty that should addressed prior to the planned test. It demonstrates the need for creating a deep understanding by relevant authorities of the relevance of testing local drugs and its implications to improve clinical care through continuous education and discussion.
		IRB members were concerned about the potential unintended consequences of providing local drugs to unstable patients	(1) Designing strict eligibility criteria to include stable patients only/patients whose condition is controlled is important (2) Strict and regular patient follow up is required (3) Data safety monitoring board need to be established
		Develop clarity of the approval process	Regulatory authority wanted application through the normal channels. That is, if the IRB indicated that regulatory authority was deemed necessary, then approval from the authority would be sought. If not thought necessary, the authority would not be approached.
Trial design, conduct and processes	Issues related to N-of-1 test	Regulatory authority members did not accept the test in principle	The need for further work to increase awareness of the potential role of N-of-1 tests in supporting the regulatory quality assurance system and improving clinical care
		A potential negative impact on the health care system if local drugs (which are cost effective) turn out to be less effective	This is a major issue. Local drugs represent an affordable option in usual care. The findings of the proposed tests may require a change from usual practice.
			Further work, including meetings and workshops that enhance understanding and benefits of the study (with the inclusion of other parties they trust), could be a possible solution. This work should emphasize (1) Short term and long term negative consequences of using medicines that are not proven to work (2) the contribution of N-of-1 tests to patient care and advancement of the health care system (3) designing strategies that can anticipate and respond to findings of the proposed N-of-1 tests are imperative, eg. a system regarding who should pay if local drugs do not work on an individual patient and the patient cannot afford alternative drugs. Also dialoguing with medicine manufacturers on the benefits of identifying the cause of the problem(s) and correcting it.
	Recruitment and retention	Doctors are too busy and lack research skill to recruit and make follow up patient assessments.	Due to high patient load and lack of research culture in hospitals, establishing an N-of-1 testing service in universities and research facilities could be a pragmatic solution. Then, provide an N-of-1 test service to the hospitals through a patient referral-feedback model, a service that clinicians are used to (referral from hospital, test, and report back to the hospital).
		Most patients are poor, so cannot afford trial related costs	Financing of N-of-1 tests deserves attention as patients alone cannot afford additional costs of these tests. Apart from the government and patient-centred care advocators, insurance and local pharmaceutical companies should be involved in this matter
		Patients are busy in their daily life	Limit patient follow up and use health technologies as much as possible in the proposed test
		Most patients are illiterate -they can’t read, or understand and record on trial documents	Recruit only patients who are literate or have a literate assistant for the duration of the study.
	Ethical and regulatory issues	Mixed views on ethical requirements	The IRB members believe that N-of-1 tests on local drugs (which are already approved) is part of quality assurance, and hence IRB approval suffices. Whereas key informants of the regulatory authority emphasised the need for regulatory approval so as to monitor potential unanticipated negative consequences of the proposed test. Posing these regulatory requirements negatively affects the feasibility of using N-of-1 tests in daily clinical care.
			These issues can be addressed by (1) including education of patients and doctors regarding interpretation and scope of individual N-of-1 study results in the proposed study as a possible option to reduce/avoid the risk of biased negative perceptions about local drugs (2) As mentioned above, meetings and workshops involving members of both IRB and regulatory authority are needed to address the tension of mixed views regarding ethical requirements and to develop a mutual guideline regarding the level of endorsement required for future N-of-1 tests on local drugs.
		Develop clarity of the approval process	The regulatory authority stated that they would only be involved in applications presented through the normal channels. That is, if the IRB indicated that regulatory authority was deemed necessary, then approval from the authority would be sought. If the IRB did not think it was necessary, the authority would not be approached.

Stakeholders from the regulatory authority did not support testing local generics in clinical settings. This was considered to be the major threat to the feasiblity of proposed research. They emphasised the potential negative consequences the test might have on patients, drug companies and the authority itself. Addressing the lack of access to affordable medicines is a major priority for developing countries like Ethiopia. Negative results from such therapeutic equivalence tests may be seen as a threat as they could lead to loss of confidence in local companies. Usual care (particularly for those who cannot afford expensive imported medicines) is dependent on local drugs and the government cannot afford to replace those drugs. In addition, pharmaceutical companies *may* bring *claims (to the authority)* against study results as their products have already approved by the authority. The regulatory authority has banned medicines in the past when strong evidence of ineffectiveness was reported from multiple sources. Hence the risk of litigation is quite low [43]. In some cases, the attitude of avoiding potential problems by not identifying them (lack of transparency) stems from a culture where it is important not to lose face [44].

The issue of how N-of-1 tests need to be treated – that is as clinical care or clinical research- at the point of care was also identified as a major threat. In contrast to other groups (who believed N-of-1 tests are at best clinical care and research tools and that IRB approval sufficed to conduct them), key informants from the regulatory authorities said that N-of-1 tests would be treated as clinical trials in Ethiopia, requiring regulatory approval and hence very onerous extra conditions prior to conducting them. This is in contrast to medical researchers who advocate the routine use of N-of-1 tests, arguing that N-of-1 tests, whilst employing randomization, blinding, and objective outcome assessment, can be considered as simply an enhanced form of clinical care, and do not automatically translate into research requiring regulatory authority approval [45].

Whether N-of-1 tests require ethical approval or not is a continuing source of debate in the literature regarding the ethics of N-of-1 tests [27, 31, 46, 47] However, requiring formal regulatory approval has not been raised as a major challenge to conducting N-of-1 tests in the literature, because N-of-1 tests are commonly used for approved therapies. The general principle is that, where the intent is to generate generalisable knowledge, IRB approval should be sought. If the intent of the N-of-1 study is to assist in the conduct of patient care, then IRB approval should not be required [27]. In a recent study on whether N-of-1 tests require IRB approval or not, more than half of the responding IRBs regarded N-of-1 tests as meeting the definition of clinical research which require IRB approval, while the other half considered them to be clinical tools not requiring ethical or regulatory oversight [47]. The idea of further regulatory approval to conduct N-of-1 tests in Ethiopia implies that the generally accepted rules relating to ethics of N-of-1 trials may not apply in the context of developing countries. In this regard, implications of the findings could also be important considerations.

We also reviewed the local literature to examine and compare these perspectives [14, 48]. We concluded that individual N-of-1 tests to prove the interchangeability of approved drugs, which are already in use by patients, largely constitutes a quality assurance activity.

In addition to the above major threats, many minor impedements to feasibility were identified. Although local drugs are approved for use by patients, IRB respondents expressed their concerns about involving patients whose blood pressure was not controlled. They stated that safeguards were important, particularly stringent oversight on their part. The main barriers reported by physicians included workload and lack of time in their daily routine. Some also mentioned lack of experience in research and research having a bad reputation. A few physicians were also concerned about the effect of the test on the physician-patient relationship, if the local drugs were shown to be less effective. Financial difficulties in covering the daily costs of living and medications, as well as lack of time to come to the hospital, were raised by patients. A lack of experience and knowledge oon the part of those people working at the regulatory level, the name ‘trial’ and the fact that the N-of-1 concept was new to the country were challenges reported by IRB members. Many of these barriers were also identified as challenges for expanding N-of-1 clinical care in developed countries [49].

Participants also suggested solutions for the above barriers to ensure the successful implementation of the study. Suggested solutions to address barriers related to physicians included; selection of interested physicians, provision of training and incentives. Participants suggested compensation for transport, creating awareness, provision of free drugs and a convenient time schedule to address challenges related to patients.

This is the first study to assess the acceptability of, using the principle of N-of-1 testing to assess therapeutic equivalence of untested generic drugs in a resource poor setting. However, the study has some limitations. Apart from the regulatory authority members, all participants were recruited from a single institute. The intention of this study was to inform the design, approval and implementation of a pilot of N-of-1 tests in Ethiopia. In this sense, as is typical of feasibility studies, internal validity was prioritised at the expense of external validity. Pre-reading material and education sessions were to familiarize participants with each other and also with the concept of N-of-1 trials (and the rationale for the proposed study), thereby facilitating active and quality group interaction and discussion about the research topic. However, the content of these educational resources and how they were delivered might have influenced views of the participants about the appropriateness of the N-of-1 trials to test local medicines. Also, the use of a male facilitator for the female patient focus group discussion, may have influenced the willingness of the participants to share their views. Female participants may have been more open and confident if a female facilitator had led the discussion. However, a reflective journal was part of the research process (including in the preparation and provision of education sessions) to reduce the risk of the researcher misleading participant expectations and beliefs. Many patients had no formal education. As there is a common belief by patients that ideas of educated people are positive and beneficial, patient participants may have been especially motivated to take part in the proposed trial. They also may have been prone to social acceptability bias in response to questions about the acceptability and benefits of the proposed N-of-1 tests. However, we did recruit from a wide range of participants to maximize representativeness of the sample.

## Conclusions

In conclusion, the study highlighted key uncertainties that impact the acceptability of using N-of-1 tests to assess the therapeutic equivalence of locally manufactured drugs in Ethiopia. Therefore, a number of changes that addressed the contextual threats and regulatory challenges identified in this research, were necessary before progressing to the conduct of the proposed study. There was a need for open, in-depth discussion of key issues in order to appropriately inform subsequent trial design decisions after this qualitative study. Finally, we acknowledge that the political will to ensure that quality becomes a top priority on the health reform agenda is also critical to improve quality of clinical care in developing countries.

## Supplementary information

**Additional file 1.** Interview discussion guide.

**Additional file 2.** Educational materials.

## Data Availability

To protect the anonymity and confidentiality of the participants, the data used (interview transcripts) are not made generally available, with the exception of the data that has been choosen for presentation in the manuscript.

## Abbreviations

AAERC: ALERT Ethics Review Commitee
AHCRQ: Agency for Healthcare Research and Quality
AHRI: Armauer Hansen Research Institute
ALERT: Africa Leprosy and Tuberculosis Rehabilitation and Treatment
FGD: Focus Group of Discussion
FHMACAE: Food, Medicine and Healthcare Administration and Control Authority of Ethiopia
IRB: Institution review board
USA: United States of America
