# Supplementary material for: Acceptability and barriers to implementation of N-of-1 tests in Ethiopia - a qualitative study
Source: BMC Med Res Methodol. 2019 Oct 15;19:192. doi: 10.1186/s12874-019-0832-7 (PMC6794767; doi:10.1186/s12874-019-0832-7)
Supplement: Supplementary file 1 — Additional file 1. Interview discussion guide. [file 12874_2019_832_MOESM1_ESM.docx]

**Additional file 1: Interview discussion guide**

1. Due to lack of pharmacokinetic bioequivalence data on locally produced medicines, this project proposed N-of-1 tests as a clinical bioequivalence to test bioequivalence (interchangeability of locally manufactured medicines) with brand name drugs containing the same medicine - so that physicians can prescribe these cheaper alternatives with confidence and patients can take them with confidence. Ultimately, thus could promote effective generic drug substitution and development of local pharmaceutical companies.

Q. What is your overall impression on the project?

Probe: Why do you think so?

Probe: What are the advantages and disadvantages?

1. N-of-1 study approaches are primarily used to improve quality of clinical care for individual patients. Similarly, in the proposed project, N-of-1 bioequivalence tests will be used to testing an approved therapy for the same indication so as to improve clinical care decision. Thus, we are considering the proposed method as clinical care tool, not as a clinical research method.

Q. What is your reaction to this?

Probe: Do you think N-of-1 tests should be considered as a research tools as well? Why?

Probe: Do you think N-of-1 tests should be considered as drug approval trials? Why?

1. Based on the guidelines and international experiences, the need for IRB approval for N-of-1 tests has been a subject of debate. Thus, we believe IRB approval would suffice for conducting the proposed N-of-1 bioequivalence tests. However, due to the scope and intention of N-of-1 studies, the need for regulatory approval has not been raised before so far.

Q. What do you think about this?

Probe: Do you think it should be exempted from IRB approval?

Probe: Do you suggest the need for regulatory approval? Why?

1. Accordingly, we expect some aspects of GCP such as regulatory approval, inspection, monitoring and auditing won’t apply.

Q. Do you agree with this?

Probe: Should all the GCP aspects apply? Which should and which shouldn’t?

1. What do you think are the barriers to conduct the pilot trial? Comment the following aspects: logistics, personnel, infrastructure and patient recruitment.
2. Would you please discuss the potential solutions for these barriers?

**CLOSING QUESTION**

We have asked a lot of question of you. Now we want to turn the tables a bit. What questions do you have of us that are related to this project?

Thank you to everyone for your efforts. With your comments we will be better able to design and implement a relevant project.
