# Supplementary material for: Acceptability and barriers to implementation of N-of-1 tests in Ethiopia - a qualitative study
Source: BMC Med Res Methodol. 2019 Oct 15;19:192. doi: 10.1186/s12874-019-0832-7 (PMC6794767; doi:10.1186/s12874-019-0832-7)
Supplement: Supplementary file 2 — Additional file 2. Educational materials. [file 12874_2019_832_MOESM2_ESM.docx]

**Additional file 2: Educational materials**

**A-Educational materiall for Patients**


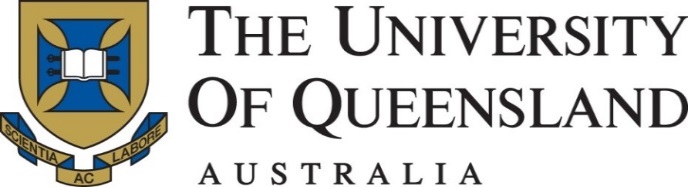
 ****

## **Topic:** Acceptability and barriers to implementation of N-of-1 trials in a resource-limited clinical setting: Ethiopia as a case study -A qualitative study

**Introduction**

Every day, millions of people are taking medications that will not help them. This happens because, in classical RCTs, scant data are collected from individual participants on factors such as genetics, lifestyles and diet, which may impact on the effectiveness of the test treatment. This leads to increased heterogeneity of treatment effects of the same drug among individual patients. Precision medicine researchers consider the many factors that shape a person's response to a particular treatment. The Food and Drug Administration (FDA) of America lists the proportions of non-responders by disease category, ranging from 38% for depression to 75% for cancer^.^ Moreover, ineffective drugs might result from lack of strong medicine regulations. Bioequivalence studies are regulatory requirements to prove quality of generic drugs. In Ethiopia, though there are strong movements, up to now, there is currently no accredited bioequivalence center. Local pharmaceutical companies are not required to present bioequivalence data. Studies conducted on drug prescribers in Ethiopia reported a negative perception of generic drugs and distrust of the local drug regulatory authorities. This may lead physicians to prescribe unaffordable costly brand drugs for patients to allay patient concerns.

**What motivates this project?**

The idea of the project was motivated by what is posted on the pharmacovigilance homepage of the Ethiopian regulatory authority. The authority posted that production of one local manufactured drug was banned upon receiving claims of ineffectiveness of a drug from various health professionals.

**About bioequivalence**

Drugs are originally produced by one pharmacutical company and are conicdered to be the perfect compator products. Following patent off period, other caompanies can produce a similar product with comparable efficy and saftey. This drugs are called generic drugs. However, before marketting, the comparative efficacy and saftey of this drugs againist ther original products should be proved using bioequivalence studies. "***Bioequivalence***" means that the active ingredient of 2 drug products has the same rate and extent of absorption. If two drugs are bioequivalent there is ***no clinically significant difference*** in their bioavailability & they are ***therapeutically interchangeable***.

**What is the aim of the project?**

This project introduce N-of-1 tests in Ethiopia for introducing personalized medicine and promoting quality use of medicines through an affordable way of proving therapeutic equivalence. N-of-1 tests are multi-cycle cross-over trials of a treatment against a comparator, all conducted within an individual patient.

**Figure 1. Example of an N-of-1 test**

Comparator drug

Comparator drug

Trial drug

Trial drug

Comparator drug

Trial drug

Pair 1

Pair 2

Pair 3

Daily symptom

assessments

Assessment of relative treatment effect for each pair

N-of-1 tests are indicated whenever there is substantial uncertainty regarding the comparative effectiveness of different treatments being considered for an individual patient. N-of-1 tests may help patients and clinicians recognize ineffective therapies, thus minimizing adverse effects, and conserving health care resources. Authors from US Department of Health and Human Services documented the pragmatic use of N-of-1 as a means to formally assess the bioequivalence of generic drugs. One such example came from Canada where a group of researchers used N-of-1 tests to prove interchangeability of generic warfarin with the brand product.The project has two phases; a first phase to conduct N-of-1 accesptablity study and a second phase to run a pilot N-of-1 test.

**What is the oobjective of acceptablity study**

The objective of this evaluation project is to assess the acceptability of the proposed test and its implementation approaches, and to explore practical barriers to the conduct of N-of-1 tests in Ethiopia and use this knowledge to inform a pilot test.

**The setting for the planned pilot trial**

We plan to conduct a pilot trial at ALERT complex. The pilot test will be supported by the University of Queensland and AHRI. The University of Queensland leads the world in exploring the use of N-of-1 tests in clinical practice. MrChalachewAlemayehu, who served as clinical trial staff in different positions at AHRI, is the lead investigator for this project. He is currently studding his PhD in medicine from UQ.

**Project implementation strategies**

In the pilot trial, we plan to test one or two locally manufactured (Cadila, Ethiopia) first-line anti-hypertensive drugs (Amlodipine and Enalpril Maleate) against brand name amlodipine and enalapril at ALERT hospital. We selected anti-hypertensive drugs because hypertension is one of the leading chronic disease in Ethiopia, and is also suitable for N-of-1 tests. Proving clinical equivalence is warranted because of uncertainty related to lack of bioequivalence data.

During the pilot trial, objectively generated data from each patint will be used to improve clinical care of indivisual patients. There fore, in this project, N-of-1 tests are presented as part of clinical care, not for research where trials are conducted as part of drug approval.

**B-Educational material for health professionals and clinical researchers**


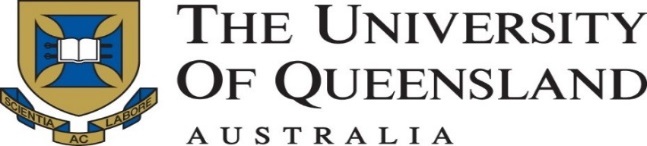
 ****

## **Topic:** Acceptability and barriers to implementation of N-of-1 trials in a resource-limited clinical setting: Ethiopia as a case study -A qualitative study

**What is the problem?**

Every day, millions of people are taking medications that will not help them. This happens because, in classical RCTs, scant data are collected from individual participants on factors such as genetics, lifestyles and diet, which may impact on the effectiveness of the test treatment. This leads to increased heterogeneity of treatment effects of the same drug among individual patients. Precision medicine researchers consider the many factors (genetic, diet and environmental, among others) that shape a person's response to a particular treatment. The Food and Drug Administration (FDA) of America lists the proportions of non-responders by disease category, ranging from 38% for depression to 75% for cancer^.^ Moreover, ineffective drugs might result from lack of strong medicine regulations. Bioequivalence studies are regulatory requirements to prove quality of generic drugs.In Ethiopia, though there are strong movements, up to now, there is currently no accredited bioequivalence center. Local pharmaceutical companies are not required to present bioequivalence data. Moreover, the country is dependent on generic drugs which are imported primarily from India. A regulatory system assessment of sub-Saharan African countries (including Ethiopia) by WHO concluded countries did not have the capacity to control the quality, safety and efficacy of the medicines circulating on their markets or passing through their territories. Some studies reported that some imported generic drugs to Africa had poor quality and other studies also identified similar problems with marketed drugs. Moreover, studies conducted in drug prescribers in Ethiopia reported a negative perception of generic drugs and distrust of the local drug regulatory authorities. This may lead physicians to prescribe unaffordable costly brand drugs for patients to allay patient concerns.

**What motivates this project?**

The idea of the project was motivated by what is posted on the pharmacovigilance homepage of the Ethiopian regulatory authority. The authority posted that production of one local manufactured drug (Niclosamide) was banned upon receiving claims of ineffectiveness of a drug from various health professionals.

There may be insufficient human resource and equipment capacity to monitor the quality of locally produced and imported drugs in poor resource settings. Neither the patient nor the country can afford the cost of poor quality drugs. Therefore, in case of uncertainties, clinicians need backup tools to objectively assess the claimed clinical equivalence of generic drugs.

**About bioequivalence**

"***Bioavailability***" refers to the rate and extent of absorption of an active ingredient from a drug product so that it becomes available at its site of action. "***Bioequivalence***" means that the active ingredient of 2 drug products has the same rate and extent of absorption. If two drugs are bioequivalent there is ***no clinically significant difference*** in their bioavailability & they are ***therapeutically interchangeable***. This means it is difficult to be certain about therapeutically equivalence of a generic drug without “bioequivalence tests”, when appropriate. As mentioned above, currently, BE report on locally manufactured drugs is not required.

**What is this project about?**

This project introduce N-of-1 tests in Ethiopia for introducing personalized medicine and promoting quality use of medicines through an affordable way of providing systematic quality assurance. N-of-1 tests are multi-cycle, double-blinded, controlled cross-over trials of a treatment against a comparator, all conducted within an individual patient.

**Figure 1. Example of an N-of-1 test**

Comparator drug

Comparator drug

Trial drug

Trial drug

Comparator drug

Trial drug

Pair 1

Pair 2

Pair 3

Daily symptom

assessments

Assessment of relative treatment effect for each pair

N-of-1 tests are indicated whenever there is substantial uncertainty regarding the comparative effectiveness of different treatments being considered for an individual patient.N-of-1 tests may help patients and clinicians recognize ineffective therapies, thus minimizing adverse effects, and conserving health care resources. Authors from US Department of Health and Human Services documented the pragmatic use of N-of-1 as a means to formally assess the bioequivalence of generic drugs. One such example came from Canada where a group of researchers used N-of-1 tests to prove interchangeability of generic warfarin with the brand product.The project has two phases; a first phase to conduct N-of-1 accesptablity study and a second phase to run a pilot N-of-1 test.

**What is the oobjective of acceptablity study**

The objective of this evaluation project is to assess the acceptability of the proposed test and its implementation approaches, and to explore practical barriers to the conduct of N-of-1 tests in Ethiopia and use this knowledge to inform a pilot test.

**The setting for the planned pilot trial**

We plan to conduct a pilot trial at ALERT complex. The pilot test will be supported by the University of Queensland and AHRI. The University of Queensland leads the world in exploring the use of N-of-1 tests in clinical practice. Mr Chalachew Alemayehu, who served as clinical trial staff in different positions at AHRI, is the lead investigator for this project. He is currently studding his PhD in medicine from UQ. His project is to introduce N-of-1 tests into his country to promote PM and improved capacity to provide drug quality assurance in his country. He is currently involved in one of N-of-1 tests being conducted in Australia. Professor Mitchell’s group at UQ, which includes Dr Jane Nikles, has accumulated more than a decade of experience in N-of-1 tests. During the actual pilot trial period, one of these experts will visit the research site to provide assistance and experience sharing.

**Project implementation strategies**

1. **Specific components of the pilot trial**

The source of drugs for such trials may range from drug vender to pharmaceutical companies. We plan to use drugs from wholesale pharmacy. The scope of these studies may range from narrow where they are be applied at the point of care to wider where the regulatory authorities make use of this tools. In the pilot trial, we plan to test one or two locally manufactured (Cadila, Ethiopia) first-line anti-hypertensive drugs (Amlodipine and Enalpril Maleate) against brand name amlodipine and enalaprilat ALERT hospital. We selected anti-hypertensive drugs because hypertension is one of the leading chronic disease in Ethiopia, and is also suitable for N-of-1 tests. Proving clinical equivalence is warranted because of uncertainty related to lack of bioequivalence data.

**2. Clinical care vs Clinical research-**

Differentiating clinical care from research employing experimental therapies can be difficult. Research means a systematic investigation, including research development, testing and evaluation, designed to develop or contribute to generalizable knowledge. Activities which meet this definition constitute research for purposes of this policy, whether or not they are conducted or supported under a program which is considered research for other purposes. In contrast, clinical care involves any decision making and practices aimed at providing individual patient service.

The distinction between Clinical Care versus Clinical Research lies in the primary objective of the n-of-1 trial. If the primary interest is to produce local knowledge to inform treatment decisions for individual patients (like which antihypertensive medicine works best), n-of- 1 trials so conducted should be interpreted as clinical care, and are not subject to clinical trial regulations for humansubjects. Alternatively, if the primary interest is to produce generalizable knowledge to inform treatment decisions for future patients, such n-of-1 tests should be interpreted as human subjects research and required to comply with the standards of such research. An example of this would be using the results of multiple N-of-1 studies to determine which pain relief for osteoarthritis works for most of a population of people.

Therefore, N-of-1 tests designed to evaluate therapeutic effectiveness in a single individual are not research. Our plan is to use this test as clinical care, not as clinical research.

**C-Material for ethical and regulatory authorities**


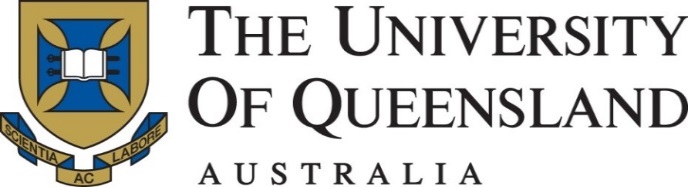
 ****

## **Topic:** Acceptability and barriers to implementation of N-of-1 trials in a resource-limited clinical setting: Ethiopia as a case study -A qualitative study

**Overall intent:** The ultimate purpose of the project is to introduce N-of-1 clinical care tools as means of practicing Patient Centred Medicine and Quality Use of Medicine. However, assessing the acceptability of their implementation strategies among relevant stakeholders are crucial before conducting the actual feasibility trial.

**Challenges in the standard medical care and the need for transition to patient centred medicine:** Both patient centred medicine (PCM) and the current standard clinical care are similar as both require data collection so as to decide the best care for individual patients. Unlike the standard care, data collection and analysis in PCM are conducted in a *scientific* way. In this transition, physicians will act as both practitioners and researchers. The medical care movement from the standard care to patient centred care in the developed countries has started because of the following reasons.

Every day, millions of people are taking medications that won’t help them well. This happens because of several reasons. First, in the routine clinical practice, physicians apply average results generated from classical Randomized Controlled Trials (RCTs). Various studies showed that heterogeneity of treatment effects (HTE) of the same drug among individual patients are very common. The Food and Drug Administration (FDA) of America lists the proportions of non-responders by disease category, ranging from 38% for depression to 75% for cancer^.^ Second, there could be uncertainties related to lack of evidence on comparative effectiveness of drugs to be considered on a particular patient. This may lead physicians to prescribe unaffordable costly brand drugs for patients to allay patient concerns. In rare cases, this may result in use of use ineffective drugs. For example, bioequivalence studies are regulatory requirements to prove quality of generic drugs. In Ethiopia, though there are strong movements, up to now, there is currently no accredited bioequivalence center. Local pharmaceutical companies are not required to present bioequivalence data. Moreover, the country is dependent on generic drugs which are imported primarily from India. A regulatory system assessment of sub-Saharan African countries (including Ethiopia) by WHO concluded countries did not have the capacity to control the quality, safety and efficacy of the medicines circulating on their markets or passing through their territories. Some studies reported that some imported generic drugs to Africa had poor quality and other studies also identified similar problems with marketed drugs. Moreover, studies conducted in drug prescribers in Ethiopia reported a negative perception of generic drugs and distrust of the local drug regulatory authorities. Therefore, ensuring quality of clinical care and enhancing routine use of cheaper locally produced drugs require an assurance that these drugs are clinical equivalence.

**What motivates the project?**

The idea of the project was motivated by what is posted on the pharmacovigilance homepage of the Ethiopian regulatory authority. The authority posted that production of one local manufactured drug (Niclosamide) was banned upon receiving claims of ineffectiveness of a drug from various health professionals.

There may be insufficient human resource and equipment capacity to monitor the quality of locally produced and imported drugs in poor resource settings. Therefore, in case of uncertainties, clinicians need backup tools to objectively assess the claimed clinical equivalence of drugs. Above all, introducing a quality assurance clinical tools are crucial for confident and wider use of locally produced drugs.

**What is this project about?**

This project introduce N-of-1 tests in Ethiopia for introducing personalized medicine and promoting quality use of medicines through an affordable way of providing systematic quality assurance. N-of-1 tests are multi-cycle, double-blinded, controlled cross-over trials of a treatment against a comparator, all conducted within an individual patient.

**Figure 1. Example of an N-of-1 test**

Comparator drug

Comparator drug

Trial drug

Trial drug

Comparator drug

Trial drug

Pair 1

Pair 2

Pair 3

Daily symptom

assessments

Assessment of relative treatment effect for each pair

N-of-1 tests are indicated whenever there is substantial uncertainty regarding the comparative effectiveness of different treatments being considered for an individual patient.N-of-1 tests may help patients and clinicians recognize ineffective therapies, thus minimizing adverse effects, and conserving health care resources. Authors from US Department of Health and Human Services documented the pragmatic use of N-of-1 as a means to formally assess the bioequivalence of generic drugs. The project has two phases; a first phase to conduct N-of-1 acceptability study and a second phase to run a pilot N-of-1 test.

**What is the oobjective of acceptablity study**

The objective of this evaluation project is to assess the acceptability of the proposed test and its implementation approaches, and to explore practical barriers to the conduct of N-of-1 tests in Ethiopia and use this knowledge to inform a pilot test.

**Project implementation strategies**

1. **Specific components of the pilot trial**

The source of drugs for such trials may range from drug vender to pharmaceutical companies. We plan to use drugs from wholesale pharmacy. In the pilot trial, we plan to test one locally manufactured drug against brand name in ALERT hospital.

**2. Clinical care vs Clinical research and clinical trial**

Research means a systematic investigation, including research development, testing and evaluation, designed to develop or contribute to *generalizable knowledge*. In contrast, clinical care involves data collection from patients with the primary intent of making the best decision for them-not primarily interested in generating *generalizable results*.

The distinction between Clinical Care versus Clinical Research lies in the primary objective of the n-of-1 trial. If the primary interest is to produce local knowledge to inform treatment decisions for individual patients (like which antihypertensive medicine works best), n-of- 1 trials so conducted should be interpreted as clinical care, and are not subject to clinical trial regulations for human subjects. In general, according to the National Institute of Health (NIH) as well as international experience, IRB approval is adequate to run N-of-1 trials for the purpose of patient care. However, as time progresses, this tests will be integrated as part of the routine clinical care.

At the pilot stage, we are planning to conduct an individual N-of-1 trial as means of proving the feasibility of conducting the test in order to make better decision for individual patients. N-of-1 tests designed to evaluate therapeutic effectiveness in a single individual are not clinical trials. However, following the pilot study, the ultimate goal is to create a platform where n-of-1 can be used to improve the quality of clinical care clinicians provide for patients, when they are feasible. In the long term, like the bioequivalence studies, drug regulatory authorities could use data from such small number of patients who are involved in N-of-1 tests (aggregated tests) to formally assess the bioequivalence of generic drugs.
